# Development and assessment of the usability of a web-based referral to treatment tool for persons with substance use disorders

**DOI:** 10.1186/s12911-021-01620-9

**Published:** 2021-09-08

**Authors:** Kelli Thoele, Mengmeng Yu, Mandeep Dhillon, Robert Skipworth Comer, Hannah L. Maxey, Robin Newhouse, Ukamaka M. Oruche

**Affiliations:** 1 Robert Wood Johnson Future of Nursing Scholar, Indiana University School of Nursing, 600 Barnhill Drive, Indianapolis, IN 46202 USA; 2 School of Informatics and Computing, 535 W Michigan Street, Indianapolis, IN 46202 USA; 3 Indiana University School of Medicine, 1110 West Michigan St., Suite 200, Indianapolis, IN 46202 USA; 4 Indiana University School of Nursing, 600 Barnhill Drive, Indianapolis, IN 46202 USA

**Keywords:** Substance-related disorder, Usability testing, Referral to treatment

## Abstract

**Background:**

Hospitalized people with unhealthy substance use should be referred to treatment. Although inpatient referral resources are often available, clinicians report that outpatient referral networks are not well-established. The purpose of this manuscript is to describe the development and usability testing of a web-based Referral to Treatment Tool (RTT © 2020 Trustees of Indiana University, all rights reserved) designed to identify treatment centers for people with unhealthy substance use.

**Results:**

The RTT was conceptualized, developed, and then populated with public use and local survey data of treatment centers from 14 market ZIP codes of hospitals participating in an SBIRT implementation study. The tool underwent initial heuristic testing, followed by usability testing at three hospitals within a large healthcare system in the Midwest region of the United States. Administrative (n = 6) and provider (n = 12) users of the RTT completed a list of tasks and provided feedback through Think-Aloud Tests, the System Usability Scale, and in-person interviews. Patients (n = 4) assessed multiple versions of a take-home printout of referral sites that met their specifications and completed in-person interviews to provide feedback. Each administrative task was completed in less than 3 min, and providers took an average of 4 min and 3 s to identify appropriate referral sites for a patient and print a referral list for the patient. The mean System Usability Scale score (M = 77.22, SD = 15.57, p = 0.03) was significantly higher than the passable score of 70, indicating favorable perceptions of the usability of the RTT. Administrative and provider users felt that the RTT was useful and easy to use, but the settings and search features could be refined. Patients indicated that the printouts contained useful information and that it was helpful to include multiple referral sites on the printout.

**Conclusion:**

The web-based referral tool has the potential to facilitate voluntary outpatient referral to treatment for patients with unhealthy substance use. The RTT can be customized for a variety of health care settings and patient needs. Additional revisions based on usability testing results are needed to prepare for a broader multi-site clinical evaluation.

*Trial Registration* Not applicable.

## Background

Over 175 million people in the world have a substance use disorder (SUD), and more than 350,000 people die each year due to substance use [1]. Additionally, more than 1.5% of the global burden of disease is caused by alcohol and drug use disorders, and alcohol use is one of the leading causes of early death and disability in males [1, 2]. In an effort to improve care for people with unhealthy substance use in the United States, the Substance Abuse and Mental Health Services Administration (SAMHSA) provided grants to study the implementation of Screening, Brief Intervention, and Referral to Treatment (SBIRT). SBIRT is an intervention that can be used in multiple healthcare settings (e.g., acute care, emergency departments, primary care) to facilitate the identification and treatment of unhealthy substance use [3]. SBIRT begins with the use of a validated screening tool to screen patients for substance misuse. Then, patients who screen positive receive a brief intervention and, when indicated, referral to treatment for long-term management of substance misuse. According to one national study of more than 17,000 patients, alcohol and drug use were significantly lower 6 months after receiving SBIRT services [3].

SUD is a chronic illness that requires proactive, long-term management [4]. Although SBIRT can address substance use, many patients who misuse substances do not receive treatment. Recent estimates suggest that only 11% of people who need substance use treatment receive care in a specialty facility, and 21.1% of untreated people with SUD report that they do not know where to get treatment [5]. This gap in treatment is due, in part, to a lack of referrals by healthcare providers. Although there are outpatient treatment centers to manage SUD long-term, healthcare providers are often not familiar with the outpatient resources available. For instance, in one study, primary healthcare providers reported that they were not equipped to address SUD at their facilities, given that they did not know where they could refer patients for ongoing management of SUD [6]. Another study found that providers thought the amount of time and resources required to determine if a patient meets eligibility criteria at a particular referral site were barriers to making referrals [7]. Although providers may identify a person with risky substance use after screening and conduct brief interventions, they may miss a critical opportunity to manage this chronic disease if they do not follow through with a referral to treatment.

Several web-based resources, managed and maintained by federal and state governments, exist to facilitate the identification of and referral to a treatment center. These tools are accessible to both patients and providers, but they have a number of limitations that impact their effectiveness. For example, SAMHSA maintains an open access substance use treatment locator on its website, allowing users to search for treatment facilities based on location, treatment type, and insurance type [8]. However, the SAMHSA treatment locator does not include specific administrative characteristics important to patients (e.g., wait times, intake processes) that may influence the accessibility of treatment services. For instance, administrative characteristics such as long wait times [9] and whether referral sites accept walk-in patients [10] may influence patients’ decisions to forego treatment. Additionally, SAMSHA and other similar tools maintained by governmental entities are generally dependent upon secondary data collected from treatment centers [11]. Yet, studies examining public sources of data related to addiction treatment and behavioral healthcare capacity cite variations in data management strategies and questionable validity and reliability [12, 13].

To address this clinical need for a resource to facilitate a referral to treatment for unhealthy substance use, we created a standardized process supported by a technology solution for use by providers. Our goal was to develop a tool that would allow healthcare providers to quickly identify appropriate referrals for substance abuse treatment facilities from a validated list of entities. Accordingly, this tool needed to provide a streamlined process to identify treatment centers using location data and patient-specific preferences and requirements. After developing the referral to treatment tool (RTT © 2020 Trustees of Indiana University, all rights reserved), usability testing was conducted with potential users to gather and evaluate data on users’ experiences (e.g., perceptions of ease of use and acceptability of a product) in order to improve product design [14] before implementation.

### Purpose

We describe the development and usability testing of this web-based RTT designed to identify treatment centers for people with unhealthy substance use. First, we describe the process of developing the RTT and then the methods and results of the usability testing of the RTT. Although integration with electronic health records and direct communication with referral treatment centers were beyond the scope of this project, they may be incorporated into the next iteration of the RTT. For the purpose of this project, referrals were limited to providing patients with printouts that contained information about the treatment center, including contact or location details, available services, and insurance/payment options. It was expected that findings will inform future tool improvements and implementation within clinical settings.

## Implementation

The study consisted of 2 phases: (1) RTT development and (2) usability testing by end users. A software application developer created the first iteration of the RTT, and then two graduate students completed heuristic testing to identify usability problems. The developer used information from the heuristic testing to create a second iteration of the tool. Next, usability testing was completed with end users (i.e., healthcare administrators and providers) and patients. End users were asked to (1) complete specific tasks using the RTT, (2) complete a survey about the usability of the RTT, and (3) answer interview questions regarding the RTT. Patients were provided with examples of referral printouts and asked to provide feedback on the content and structure of the printouts. This study underwent ethical review by the Indiana University Institutional Review Board and met the criteria for approval (#1903172112).

### Development of the referral to treatment tool

#### First iteration

The objective of developing the first iteration was to create the web-based RTT for applications on mobile devices, tablets, and desktop computers. Because healthcare providers have reported that identifying referral sites can be time-consuming and a barrier to referral to treatment, user interface planning focused on developing a simple interface that would require very few steps to find a treatment center recommendation. We began by developing a user interface to allow providers to enter patient requirements (e.g., location, treatment type) using the data items from the SAMHSA data set. This set of patient requirements would be used for filtering the list of treatment centers to include only those that matched the patient’s needs. Once providers entered patient requirements, they would see the primary information page with a resultant list of treatment centers, sorted in proximity order, either from the user’s location or from a ZIP code supplied by the patient. Providers could then select a treatment center from the list to see a “detail” page with information about the selected treatment center. This detail page would display contact information, location information (including a map display), and a list of all services, therapies, ancillary services, and payment options at the treatment center. The data for the RTT originated from the SAMHSA National Survey of Substance Abuse and Treatment Services (N-SSATS) database [15]. This database contains a listing of treatment centers, contact information, services, and payment options available at treatment centers, and the RTT was populated with the treatment centers within the 14 market ZIP codes of participating hospitals. The authors conducted telephone surveys of treatment centers to validate the N-SSATS data and identify additional characteristics of treatment facilities associated with patient accessibility. While the results of the telephone survey were largely consistent with the data obtained from SAMSHA, we also found discrepancies. If the information from SAMSHA and the surveyed treatment center differed, we used the information from the telephone survey in the RTT because it was collected more recently and in a manner similar to which a referred patient might obtain the information [16].

Once the first iteration of the tool was completed, two co-authors—with expertise in informatics and computing—completed a heuristic evaluation. The evaluators used a 35-item checklist to identify usability problems related to the appearance, content, navigation, efficiency, and functionality of the RTT. Each item was rated as either acceptable (i.e., a star or a checkmark) or unacceptable [17, 18]. After the co-authors completed the heuristic review, the ‘unacceptable’ items were discussed in detail. Namely, (1) users could not navigate backward or undo and redo actions, (2) the list of treatment centers was not sorted in any particular order, and (3) there was poor visibility of the list as users needed to scroll to view items. The results of the heuristic evaluation were shared with the research team, and investigators also identified a need to adapt and customize the RTT to different organizational contexts. Additionally, the research team aimed to create a printout that decreased patients’ burden and increased patients’ accessibility. Consequently, the team recommended creating multiple versions of the printout to determine the level of details and number of treatment centers patients preferred.

#### Second iteration

After completing the heuristic evaluation, a second iteration of the tool was produced to improve usability. We added an administrative interface that would allow hospital administrators to customize the tool for their organization. The administrative interface included methods for defining a hospital system and its associated locations, creating user accounts linked to hospital system locations, editing the SAMHSA data items included in the patient requirements settings, and editing which recovery centers would appear on the main list. By adding these functions, administrators could simplify the items displayed on the patient settings form, reducing the number of items to only those considered relevant. While the administrative functions were complex, they simplified the use of the RTT for providers.

During the development of the second iteration of the RTT, other minor user interface items were added, including a search feature, which filtered the list by the recovery center name, and a button that reset the list for a new patient, removing any previously entered patient requirements or ZIP code locations. Additionally, the printout for the referral was modified to provide different options for the information included in the printout.

**Fig. 1:**
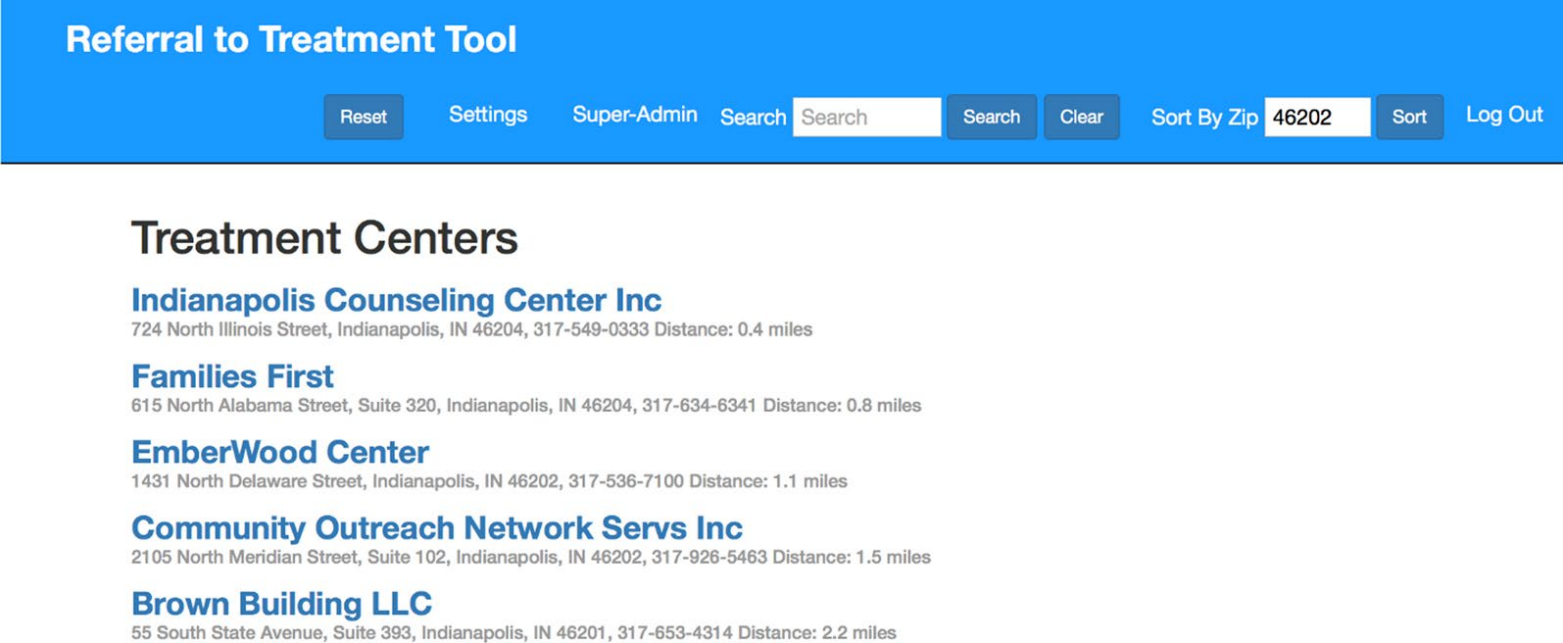
Screenshot of the RTT

**Fig. 2:**
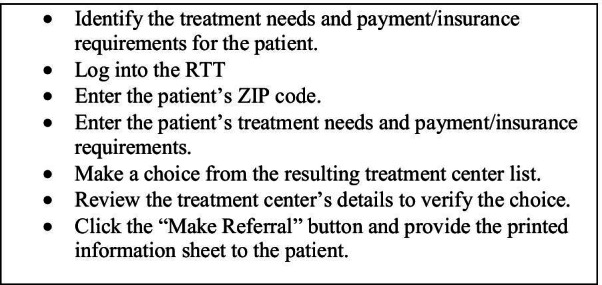
Steps to use the RTT to identify a treatment center

**Fig. 3:**
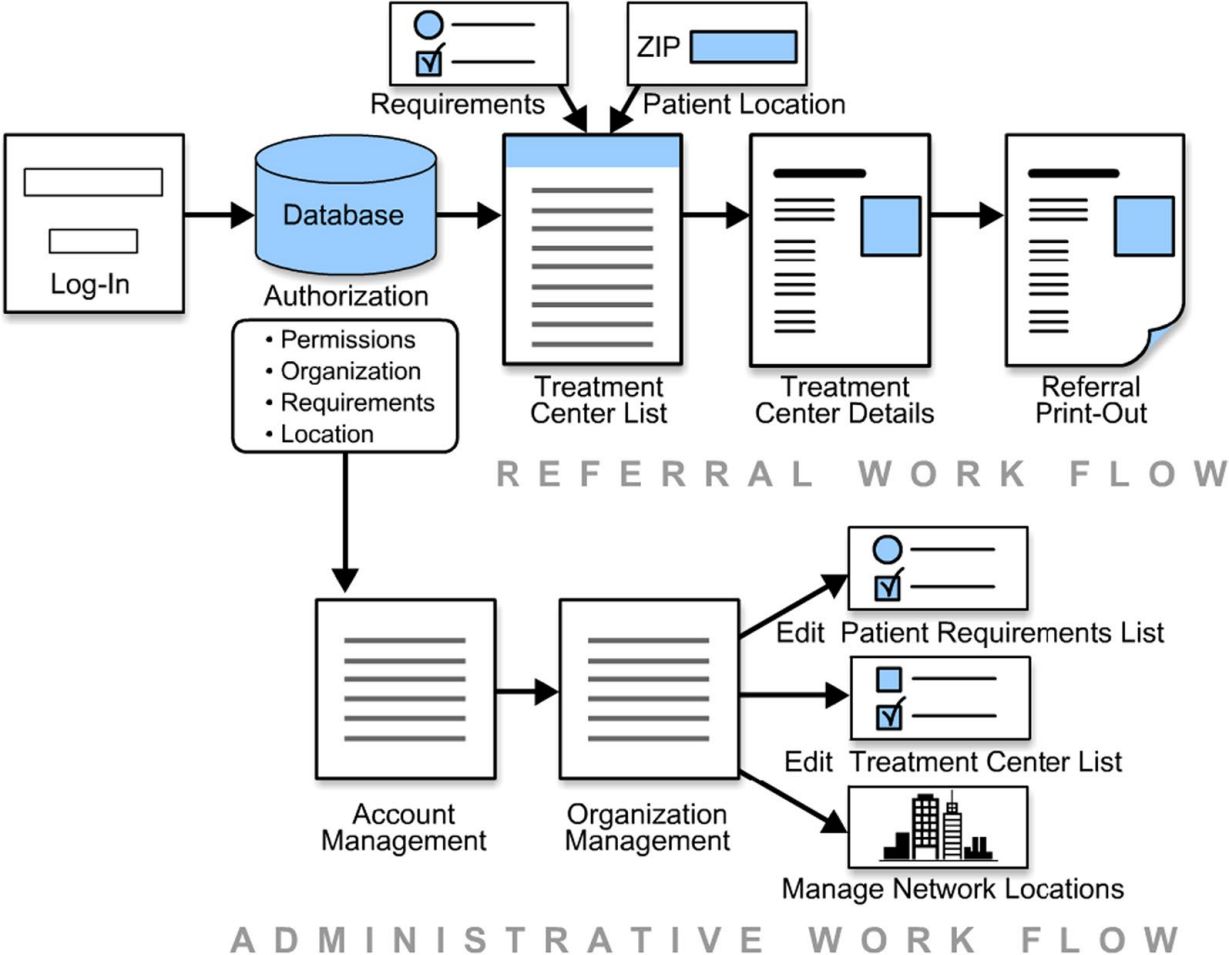
Referral and administrative work flows of the RTT

The final version of the RTT for usability testing provided a configurable tool for healthcare providers, which had many options for administrative users but was simple and streamlined for healthcare providers to use. Figures 1, 2, and 3 demonstrate an example results list and the steps to make a referral tailored to the patient’s needs using the RTT.

### RTT workflows

Workflow for the RTT (See Fig. 3) begins with the login step, which identifies the user’s role and location. For provider users, the process begins by entering the patient’s requirements and location (which defaults to the provider’s location). The resulting list of referral facilities is filtered by the requirements and ordered by proximity to the location. When a referral facility is selected from the list, detailed information about the facility is displayed on the next screen. The provider can either make the referral or return to the original list. If a referral is made, a printout of the referral facility’s location and contact information is generated. Administrative users can manage the organization’s user accounts or manage the organization’s general information, including, but not limited to, options in the patient requirements list, the list of acceptable referral facilities, and the list of locations for affiliated hospitals and clinics (Fig. 3).

### Usability testing of the referral to treatment tool

#### Design

Usability testing is a method in which data is collected while users perform tasks using a product under controlled conditions [14]. To test the usability of the RTT, administrative and provider users completed tasks using patient scenarios. Data about the usability of the RTT were collected using the Think-Aloud Test [19], System Usability Scale (SUS) [20], and semi-structured interviews. Patients received examples of printouts for treatment sites from the RTT and provided feedback via semi-structured interviews.

#### Setting and sample

Usability testing was conducted at three hospitals within a large healthcare system in the Midwest region of the United States. These hospitals were selected to obtain feedback from people across urban, rural, and suburban settings. Inclusion criteria were employees and patients/family members at one of the included hospitals. Based on the anticipated use of the RTT, participants for this study included administrative users (i.e., people who would manage users and activate/deactivate certain functions to tailor the application to the setting), provider users (i.e., physicians, physician assistants, advanced practice registered nurses, nurses, social workers, and case managers), and patients (i.e., people who would receive referrals from the tool, or their family members). Patient and family member participants were not required to have a history of substance use to participate. To participate, patients or family members had to be alert, oriented, and not in acute distress.

A nurse leader at the healthcare system identified one employee (i.e., a facilitator) at each hospital to facilitate the usability testing by recruiting participants, reserving rooms, and coordinating participants’ schedules. The investigators met with each facilitator to discuss the goals of the usability testing and the desired participants. The facilitator then identified a convenience sample of co-workers who might be willing to participate in the study as administrative users or provider users and sent them a recruitment e-mail. All individuals who were willing and able to participate then worked with the facilitator to schedule a time to participate in the usability testing. On the day of the usability testing at each hospital, the facilitator talked to charge nurses to identify patients currently admitted to the hospital who might be willing to participate. An investigator reviewed a study information sheet with all participants and answered any questions prior to participation in the study. After completing the usability testing, each participant received a $10 gift card.

### Usability testing and data collection

Usability testing was completed in one day at each site. For administrative and provider users, testing was conducted with only one participant at a time in a private conference room. Administrative and provider users individually completed the testing with investigator-provided computers. For patients, testing was conducted in their private hospital rooms or a private conference room.

#### Administrative users and provider users

**Fig. 4:**
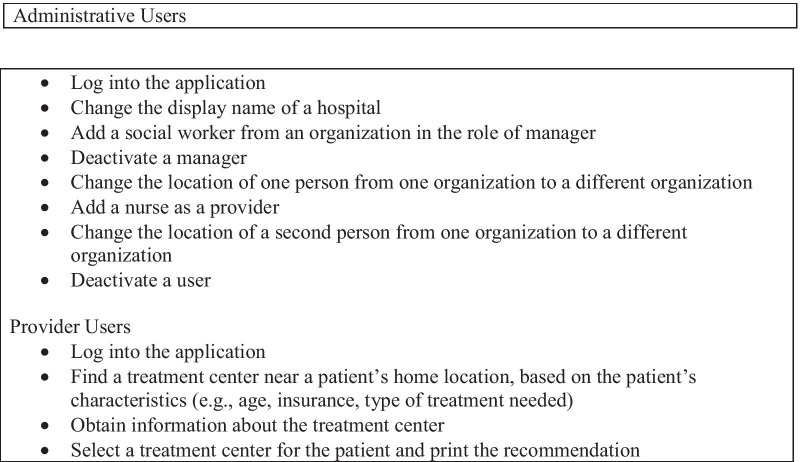
Tasks completed by administrative and provider users

The administrative and provider users were asked to perform different tasks according to their roles (See Fig. 4). The administrative users received an administrative account to log in to the website and performed tasks of a manager in the hospitals, such as adding or deleting users or updating organizational information. The provider users logged into an ordinary user account and made a referral based on a case scenario provided during the test. Participants read the tasks and case scenarios provided in a test packet and completed all the tasks using the RTT.

Several measures were used to evaluate the usability of the tool. The first was the Think-Aloud Test which is used to identify problems that arise while completing tasks [19]. Participants were asked to think and speak aloud while completing the assigned tasks, indicating which tasks they were completing and how they felt about completing each task. Each administrator completed eight tasks, and each provider user completed four tasks as detailed in Fig. 4. All sessions were audio and video recorded for data analysis.

Following the Think-Aloud Test, researchers gathered data on users’ assessment of the overall experience using the SUS [20], a standardized usability-assessment scale. The SUS questionnaire is composed of ten questions with a mix of positive and negative questions. For each question, the user rates subjective assessments of usability using a Likert scale from 1 (strongly disagree) to 5 (strongly agree) [20, 21]. To calculate the overall SUS score, we used Eq. 1, where ${U}_{n}$ refers to the rating of the ${n}^{th}$ question [22]. The minimum and maximum SUS scores are 0 and 100, respectively, where higher values suggest higher user satisfaction and a passable score is above 70 [23].

1 $$SUS=2.5\times \left[\sum \limits_{n=1}^{5}\left({U}_{2n-1}-1\right)+\left(5-{U}_{n}\right)\right]$$

After the administrative and provider users completed all tasks, the Think-Aloud Test, and the SUS, investigators used a semi-structured interview guide to obtain additional feedback. Questions included: (1) “Which part of this web application is good and why?” (2) “Which part of this web application is bad and why?” and (3) “Give your suggestions to improve the web application.” All interviews were audio-recorded.

#### Patients

Because patients would be the recipients of the information generated by the RTT, patient feedback was necessary to assess the layout and content of the printout. Patients received four different printouts from the tool at the beginning of the interview session, and patients were permitted to review the printouts for as long as necessary to become familiar with the content. One printout included detailed information (e.g., referral center, contact information, the reason for the referral, types of treatment offered at the referral center, types of insurance accepted at the referral center, wait times, intake procedures) about multiple treatment centers. The second printout included basic information (i.e., referral center name, location, and contact information) about multiple treatment centers. The third printout had basic information from only one treatment center, and the fourth printout had basic information from 2 treatment centers. Investigators used a semi-structured interview guide to obtain patient feedback, and questions included: (1) “What information is helpful to have?” (2) “How much information is relevant to you?” (3) “How many treatment centers should be included on the printout?” and (4) “Is anything missing from this information?” All interviews were audio-recorded.

### Data analysis

#### Administrative and provider users

All quantitative data were stored and analyzed using R Studio and Microsoft Excel. Investigators reviewed the audio and video recordings from the Think-Aloud Test and used a timer to measure the amount of time it took each administrative and provider user to complete each assigned task. The investigators then calculated the average time for each task among all participants.

The sample scores of the SUS were evaluated first for its normality using the Shapiro–Wilk test. Then, the difference between the observed mean SUS score and the passable score of 70 was evaluated with a *t*-test.

All interview recordings were transcribed, and then two independent reviewers used deductive content analysis to assign codes to each text unit using ATLAS.ti. After independently assigning codes, reviewers compared results and reached a consensus for the final codes. A word count method was then used to quantify the number of times each code was used in response to questions regarding the usability of the RTT.

#### Patients

Similar to the analysis of the interviews for the administrative users and provider users, patient interviews were transcribed, and then two independent reviewers used deductive content analysis to assign codes using ATLAS.ti. The final codes were then used to summarize patients’ responses to the interview questions.

## Results

Table 1 shows the type of users and their locations. A total of 22 people participated in the study, including 6 administrative users, 12 provider users (including physicians, nurses, and social workers), and 4 patients. Each session (which included the Think-Aloud-Test, SUS, and the interview) with administrative and provider users lasted between 15 and 45 min. The patient interviews lasted for 5–20 min.

**Table 1:** Participants

Location	Administrative users	Provider users	Patients
Community hospital in suburban area	1	2	1
Critical access hospital in rural area	4	6	1
Academic health center in urban area	1	4	2

### Administrative and provider users

#### Think-aloud tasks

During the Think-Aloud Test, users stated that it was easy to perform tasks, and the user interface was intuitive and easy to use. All users performed the tasks in a good mood with a positive response. None of the users showed negative emotional responses. Administrative tasks took from 2 s to 2 min and 41 s to complete (Table 2). On average, the total time it took to identify a referral site and print a recommendation for the patient was 4 min and 3 s (Table 3).

**Table 2:** Time to complete administrative tasks

Task	Average time to complete task
Log into the application	28 s
Change the display name of a hospital	1 min and 41 s
Add a social worker from an organization in the role of manager	1 min and 36 s
Deactivate a manager	2 min and 41 s
Change the location of one person from one organization to a different organization	3 s
Add a nurse as a provider	1 min and 27 s
Change the location of a second person from one organization to a different organization	2 min and 6 s
Deactivate a user	2 s

**Table 3:** Time to complete referral by provider

Task	Average time to complete task
Log into the application	29 s
Find a treatment center near a patient’s home location	3 min and 5 s
Obtain information about the treatment center	14 s
Select a treatment center for the patient and print recommendation	15 s
Total time to identify a referral site and print recommendation	4 min and 3 s

During the Think-Aloud Test, the administrative and provider users also indicated that it would be helpful to make a few changes to improve the usability of the RTT. Specifically, administrative users noted that there should be an option to delete old data and that it would be helpful to receive a confirmatory message after completing tasks (e.g., “manager deactivated”). Provider users indicated that the nav-bar user interface should include selection buttons for ZIP code, sex, payment method, and type of care. Additionally, provider users recommended that the “Setting” selection should be changed to “Filter” or “Patient Details.”

#### System Usability Scale (SUS)

The SUS questionnaire had good internal consistency (Cronbach’s α = 0.79). The SUS scores were computed for the overall group (i.e., administrative and provider users) and administrative and provider users separately. The Shapiro–Wilk test showed that the mean SUS scores were normally distributed (*p* = 0.45). Therefore, the overall mean SUS scores (M = 77.22, SD = 15.58) were used in a one-sample *t*-test to gauge whether the mean population SUS scores were greater than a passable score of 70 [20].

Table 4 shows the results of the SUS. With an overall mean of 77.22 (SD = 15.57), participants as a whole had SUS scores significantly greater than the industry passable score of 70 [t(17) = 1.74, *p* = 0.03]. Upon examining different types of users separately, provider users’ SUS scores (M = 76.25, SD = 9.20) were significantly greater than the passable average of 70 [t(11) = 2.35, *p* = 0.02], but administrative users’ SUS scores (M = 79.16, SD = 25.13) were not [t(5) = 0.89, *p* = 0.21]. A *t*-test showed that there was no difference between administrative and provider users’ SUS scores (*p* > 0.05).

**Table 4:** Results of System Usability Scale (SUS)

SUS Questions	Administrative Users (mean/std)	Provider Users (mean/std)	Overall (mean/std)
Q1 I think that I would like to use this website frequently	3.83/1.47	4.5/0.67	4.28/1.02
Q2 I found this website unnecessarily complex	2/1.55	2.17/1.11	2.11/1.23
Q3 I thought this website was easy to use	4/1.26	3.83/0.83	3.89/0.96
Q4 I think that I would need assistance to be able to use this website	2.5/1.76	2.17/1.4	2.28/1.49
Q5 I found the various functions in this website were well integrated	4.5/0.55	3.58/1.08	3.89/1.02
Q6 I thought there was too much inconsistency in this website	2/1.67	1.67/0.65	1.78/1.06
Q7 I would imagine that most people would learn to use this website very quickly	4.67/0.52	4.33/1.15	4.44/0.98
Q8 I found this website very cumbersome/awkward to use	1.5/0.84	1.83/0.83	1.72/0.83
Q9 I felt very confident using this website	4.33/0.82	3.92/0.9	4.06/0.87
Q10 I needed to learn a lot of things before I could get going with this website	1.67/1.21	1.83/0.94	1.78/1
Total SUS Score	79.17/25.13	76.25/9.2	77.22/15.57
t-score	0.89	2.35	1.97
*p*-value	0.21	0.02*	0.03*

SUS scores were compared to the industry passable standard (70). The range of scores was 0–100, where higher scores indicated the RTT was more user friendly

*Statistically significant difference between observed scores and the industry passable score

#### Interviews

Table 5 shows the codes and exemplar quotes that emerged from the administrative and provider interviews. Eleven codes were identified, and the most frequent codes were “better,” “easy,” and “good.” Administrative and provider users stated that the RTT was a “better” resource than what they were currently using in practice. Additionally, users reported that the RTT was a good and useful resource that was intuitive and easy to use. Although users generally felt favorable about the RTT, they also suggested changes to improve the RTT. For example, some button labels, particularly the “Setting” button (which opened the “Patient Requirements” settings) was confusing to users, and they recommended changing this label to something more descriptive. Also, the search function did not behave the way users expected it would. The search feature was designed to identify referral resources by ZIP code. However, providers expected the RTT to yield results if they searched for a specific healthcare system or entered a keyword associated with a known treatment center. Although the RTT provided results (i.e., a list of treatment centers) when providers searched using ZIP codes, the RTT did not provide results when providers entered other search terms.

**Table 5:** Codes and examples

Code	Example
Better	This tool is better, because the centers are listed based on distance from the patient
	This is a better source of information
Easy	It is very easy to use
	The web tool is easy to use. There is not much training required to use the tool
Good	It is a good tool. The tool has all the information required
	It is a good portal. It is much needed
Insurance	Insurance information should be in the filter to find the center
	Insurance information in center details is missing, should be there as it is big factor in decision making
No issues	There are no issues
	There is no issue, no suggestions for improvement
Old data	The data is old
	The information about this center is old data
Payment	The payment method is very important in making decisions
	Payment method should be on the front screen
Quick	The tool is quick to navigate
	It is quick to use
Search function	The search is not working. The search function should be able to search by keyword
	Search is not working well and should be optimized
Setting issue	Setting is not working, it has an issue
	The setting word is misleading, and it should be changed
Useful	It is very useful, a very good source
	Very useful to find a center near the patient

### Patients

#### Interview

Patients identified aspects of the printout and referral process that would facilitate referral to treatment. Patients generally preferred more detailed information on the printout, although one person stated that limited information is preferred if ‘the patient wants to hide information about treatment’ from his/her family. Second, a few patients mentioned that family members are an important part of the referral process, as family provide support. Regarding the actual printout, patients reported that information about insurance accepted at each treatment center was important, because ‘it takes a lot of effort to call and check if each place takes a particular insurance.’ In general, patients preferred that the printout include a map of the treatment center to make it easier to find the treatment center, and most people preferred 2–3 centers on the printout, because ‘sometimes there is a waiting period at one place.’

## Discussion

Most people with SUD do not receive treatment at a specialty facility, and one of the major barriers to referral to treatment is identifying appropriate referral sites. This article describes the development and usability testing of the RTT to facilitate referral to treatment. Participants in this study felt that the tool was useful, and they provided suggestions to improve the usability of the tool and the patient printout.

The RTT tool developed and tested in this study included information that is publicly available on SAMSHA. During the development phase of the RTT, treatment facilities were surveyed to verify the information and identify additional characteristics that may influence the accessibility of treatment for referred patients [16]. Results from the survey demonstrated variations between selected information maintained by SAMSHA and those reported by facilities, including the availability of certain services such as care coordination or Medication Assisted Treatment. Additional research is needed to identify the reasons for these variations.

By using the Think-Aloud-Test, SUS, and interviews to obtain meaningful insights from end users and patients regarding the RTT, investigators identified several actionable steps to improve the usability of the RTT. For example, participants felt the settings function was confusing and the search function did not perform as expected. Additionally, the "settings" button could be relabeled as "patient requirements" to clarify that users should enter information related to patients' needs, and the search function could be modified by optimizing the algorithm to identify relevant information using language in the search query that reflects language used by providers. In addition, when queries yield "no results," the RTT could display related results, suggestions for improving queries, or contact information for help. Lastly, given that expert clinicians have identified administrative and clinical characteristics that influence referral accessibility and appropriateness (e.g., intake processes, patient engagement, integration of services) [10], information on these characteristics could be included in future iterations of the RTT. Similar to this study, other literature supports the use of Think-Aloud-Tests and the SUS to enhance the usability of software applications [24, 25], although asking participants to think aloud while completing a task may negatively affect task performance [26].

Because over 35,000 people die each year due to substance use [1], reducing overdose deaths has become a leading health indicator [27]. The U.S. Preventive Services Task Force (USPSTF) identified substance use as a high priority, including the need for assessment, screening, and referral for drug use [28]. Processes such as SBIRT require system infrastructure to support interdisciplinary efforts so that failures or gaps in care do not occur. While the RTT provides an initial step toward eliminating gaps in care, additional work is needed to help providers and patients identify appropriate treatment centers, namely integrating quality metrics, facilitating direct communication, and maintaining updated information in the RTT.

For this study, a survey was used to verify and gather updated information. While successful, the resources required to survey treatment facilities may not always be available for future updates. Operationalizing the collection of this information in a cost-effective manner will be critical to maintaining the integrity of the information in the RTT. Several strategies have been considered. First, treatment centers can subscribe to and update their information in a database. For instance, one inpatient addiction treatment referral tool relies on facility-based subscriptions to maintain up-to-date information (https://openbeds.net/). This strategy places the onus of updating treatment center information on the centers and requires that they subscribe to be listed in the tool. This strategy assumes that the benefit of being listed in the tool outweighs the subscription cost and the amount of time it takes to update information. The long-term effectiveness of this strategy for outpatient addiction treatment centers is unclear. Another option may be to partner with state government entities that regulate treatment facilities. These entities generally require facilities to submit information on a routine basis for monitoring purposes. Potential may exist for gathering information useful for informing RTT decisions as part of these processes.

### Future iterations

The RTT is critical to clinical processes as it locates treatment options aligned with patients' and healthcare providers' preferences. Usability test results indicate that administrative, provider, and patient users found the RTT useful, though they also recommended modifications. Future research focus include (1) revise the RTT per participants' suggestions, (2) conduct usability testing on iterations incorporating additional features (e.g., quality metrics, intake processes), (3) seek partners and funding to expand the state-based survey of treatment centers, and (4) embed the tool into the SBIRT process on medical-surgical units to assess its impact on improving the incidence of outpatient referrals to treatment. Our next step in evaluating the RTT is to embed the tool into the SBIRT process on medical-surgical units in a multi-site dissemination study to improve its impact on the incidence of outpatient referrals to treatment. The team will also review the survey data to determine if the characteristics of treatment centers need to be refined, and seek partners and funding to expand the state-based survey of treatment centers.

## Conclusions

A new web-based tool, the RTT, was developed and evaluated to facilitate referral to treatment centers for people with unhealthy substance use. The RTT allows administrators to customize the tool and healthcare providers to enter patient-specific information and identify treatment centers that provide services in alignment with the patient’s needs. Administrative users and provider users reported that the RTT was useful, and patients preferred printouts with multiple treatment center options. Future research should evaluate and refine the RTT in multi-site studies to update and improve the utility and function based on patients’, providers’, and administrator users’ needs.

## Data Availability

The web-based Referral to Treatment Tool (RTT © 2020 Trustees of Indiana University, all rights reserved) is still under development; however, people interested in using the RTT can obtain access to the tool by contacting Robin Newhouse at newhouse@iu.edu. Project Name: Referral to Treatment Tool (RTT). Project Home. Page: https://comet.soic.iupui.edu/rcv. Operating System(s): Apache/Linux. Programming Language: MySQL, PHP, HTML 5, Javascript/jQuery/Bootstrap. Other Requirements: PHP 7.0 or higher, MySQL 5.6 or higher, Chrome Browser recommended. License: The MIT License, © Trustees of Indiana University. Any restrictions to use by non-academics: Use by permission.

## Abbreviations

HER: Electronic health record
IU: Indiana University
SAMHSA: Substance Abuse and Mental Health Services Administration
SUS: System Usability Scale
